# Supplementary material for: Acceptability of mental health photovoice research with adolescents in rural Mexico
Source: Glob Ment Health (Camb). 2025 Oct 24;12:e128. doi: 10.1017/gmh.2025.10080 (PMC12641300; doi:10.1017/gmh.2025.10080)
Supplement: Chatham et al. supplementary material 1 — Chatham et al. supplementary material [file S2054425125100800sup001.docx]

**Supplementary Material.** Topics Covered and Activities Facilitated During Photovoice Workshops

| **Workshop 1 / Day 1: Framing** |
| --- |
| - Overview of the project |
| - Free listing activity: What comes to mind when you hear MH and EW? |
| - MH true or false |
| - Story telling through photography, photography ethics and safety |
| - Photography prompts; identify potential pictures activity |
| - Small groups brainstormed potential pictures |
| - Photography / camera technique |
| - Distributed cameras |
|  |
| **Workshop 2 / Day 2: Collecting and analyzing** |
| - Participants brought the first round of photos to workshop |
| - SHOWED method |
| - In small groups, participants shared their pictures and began writing about them using SHOWED |
| - Reviewed preliminary answers to prompts and discussed gaps to be addressed in next round of photos |
| - Appreciative inquiry group discussions |
|  |
| **Workshop 3 / Day 3: Collecting and analyzing** |
| - Participants brought the second round of photos to workshop |
| - In small groups, participants shared their pictures and continued writing about them using SHOWED |
| - Pile sorts coding |
| - Reviewed preliminary answers to research questions and discussed gaps to be addressed in next round of photos |
| - Exhibition planning |
|  |
| **Workshop 4 / Day 4: Analyzing, reporting, and celebrating** |
| - Participants brought third round of photos to workshop |
| - In small groups, participants shared their pictures and finalized their narratives |
| - Participants finalized grouping their photos and narratives in common themes |
| - Set up for exhibition |
| - Present findings to the school community and family members |
| - Celebrated the completion of the workshops with refreshments and distribution of certificates |
