# Supplementary material for: Acceptability of mental health photovoice research with adolescents in rural Mexico
Source: Glob Ment Health (Camb). 2025 Oct 24;12:e128. doi: 10.1017/gmh.2025.10080 (PMC12641300; doi:10.1017/gmh.2025.10080)
Supplement: Chatham et al. supplementary material 2 — Chatham et al. supplementary material [file S2054425125100800sup002.docx]

Participants’ quotes in Spanish (original language) are listed in the order they appear in the manuscript.

**Learning About the Meaning, Nature, and Experiences of MH and EW**

- “(El bienestar) es nuestras emociones, es lo que te hace Feliz” (middle schooler)
- “No más le tomaba yo fotos porque me gustaban y ya, pero sino hasta después cuando el taller y pidieron las fotos que uno mismo nos gustaban, ahí es donde aprendí que por qué tomé esas fotos de los atardeceres, de los paisajes. (…) comprendí que cada momento, cada objeto, cada animal (…) es bonito, te comprende, te relaja, te trae tranquilidad” (high schooler)
- “estar con una mascota, un amigo, salir a lugares especiales, bonitos o ver fotos” (middle schooler)
- “aprendí a aceptar la separación de mis papás y a saber entenderlos y apoyarlos en lo que decidan hacer” (high schooler)
- “gracias al taller fotovoz aprendí a valorar las cosas que parecen simples y a relacionar a estas mismas con un aspecto en mi vida que para mí es importante, contribuyendo a mi salud emocional” (high schooler)
- “yo aprendí muchas cosas en este proyecto de fotovoz: que mi comunidad es hermosa y debemos de cuidar nuestras tradiciones de nuestra cultura, y la bella vista que tenemos, y la naturaleza, y las diferentes especies de animales” (middle schooler)
- “porque aquí no hay ni con quien platicarlo, no hay cómo distraerse o así. Como que sí hace falta más apoyo, ya sea en la comunidad, ya sea aquí en la escuela” (high schooler)

**Enjoying Relationships, Novelty, and Fun**

- “Me gustó convivir con las personas que vinieron, conocerlas, aprender con ellas” (middle schooler)
- “me gustó porque me sentía muy desanimado. Llegaron otras personas, las conocí, como que sentí felicidad. Yo pienso porque ya tenía mucho tiempo sin salir” (high schooler)
- “fueron amables, nos llevamos bien, nos respetamos, nos entendían, nunca nos ofendieron” (high schooler)
- “lo que más me gustó fue su alegría que todo el tiempo sonrisa, su tranquilidad, su paciencia” (high schooler)
- “ellos nos dieron confianza porque nos prestaron la cámara para sacar fotos y explicamos algunas fotos y fue una sensación bonita y confiable” (high schooler)
- “disfrutar de momentos con nuestros amigos” (middle schooler)
- “me gustó cuando salíamos hacer trabajos con compañeros” (high schooler)
- “ya tenía tiempo que no salía de mi casa. Sí salía, pero de la tienda a mi casa y de la casa esta, antes no—ya hace mucho tiempo que no iba a ver” (middle schooler)
- “me gustó y me impresionó de saber cámaras que ya imprimen las fotos. Solo esperé unos minutos y ya” (middle schooler)

**Wishing for More Time, More Play, and Continuity**

- “Me gustaría más seguir sacando más fotos y seguir más con el proyecto de fotovoz” (high schooler)
- “el taller debería incluir también técnicas de fotografía y ponerlas en práctica” (high schooler)
- “que vengan más seguido, que den talleres nuevos, como, hablar de la depresión” (middle schooler)
- “que nos hicieran sentir más en confianza” (high schooler)
- “más información (sobre) en donde dan información” (high schooler)
